# Lipidomics: The Function of Vital Lipids in Embryogenesis Preventing Autism Spectrum Disorders, Treating Sterile Inflammatory Diatheses with a Lymphopoietic Central Nervous System Component

**DOI:** 10.1155/2011/137175

**Published:** 2010-12-20

**Authors:** Thomas Tallberg, Jan Dabek, Raija Hallamaa, Faik Atroshi

**Affiliations:** ^1^Helsinki Institute for Bioimmunotherapy, Ltd, 00200 Helsinki, Finland; ^2^Department of Pharmacology, University of Helsinki, Helsinki, Finland

## Abstract

The central role performed by billions of vital central nervous system (CNS) lipids “lipidomics” in medical physiology is usually overlooked. A metabolic deficiency embracing these vital lipids can form the aetiology for a variety of diseases. CNS lipids regulate embryogenesis, cell induction, mental balance by preventing autism spectrum disorders, depression, burn-out syndromes like posttraumatic stress disease PTSD, by guarding normal immunity, treating sterile inflammatory diatheses with a titanium containing lymphopoietic CNS lipid component. The propaganda driving for unphysiological fat-free diets is dangerous and can cause serious health problems for a whole generation. This article presents a broad list of various mental and motor bodily functions of which the healthy function depends on these vital CNS lipids. A rigorous fat-free diet can provoke these metabolic lipid deficiencies but they can fortunately be compensated by dietary supplementation, but not by pharmacologic treatment.

## 1. Introduction

The most important bodily components involved in preserving health and normal cell induction embrace the very numerous “vital lipids” in our central nervous system (CNS). These consist of a multitude of lipids complexes formed with proteins, sucker moieties, neutral lipids with amino groups, and metal trace-element ions, forming a wide variation with unsaturated to poly-unsaturated lipid moieties. The extensive CNS inductional actions ruled by these lipids and organ-specific mitochondria comprise embryogenesis, tissue-specific induction, mental and motor balance. Its functions are here depicted as, “*lipidomics*” which together with *proteomics, genomics*, and organ-specific *mitochondria* regulate all our cells and bodily missions. A deficiency comprising vital central nervous lipids (CNS- lipids) can luckily be compensated by dietary supplementary meanss and thus alleviates disparate ailments caused by such metabolic lipid deficiencies. The cause of these health functions is very complex and, listed in Table 1. Some of these disease entities spring from a crucial lack of a lymphopoietic CNS component which is necessary for a normal immune response. If this titanium-containing vitamin-like substance is lacking, it may lead to various sterile inflammatory diatheses, Table 2. Only some aspects of the “lipidomics” leading to these lipid deficiency diseases will be discussed in this paper.

## 2. Autism Seems to Stem from a Lack of Maternal Circulating Neurogenic Lipids during Gestation

The perplexing multitude of clinical signs and symptoms in patients with autism spectrum disorders (ASDs) has naturally led to the misconception that there must be multiple etiologic factors involved. The whole problem is eminently reviewed by, Cuccaro et al. [2].The most recent hypothesis is that autism is caused by industrial chemicals [3]. It could be true for some clinical cases but could not explain the recent observations with an increased incidence in the whole western world. 

A plausible explanation for the aetiology of autism and ADHD can clinically be modulated by intake of CNS-lipids. The concurrent increase in adults suffering from associated neurological ailments like depression, insomnia, hyperaesthesia, and pain is that these chronic neurologic disorders could be caused by a deficient dietary content of vital lipids, or linked to a depressed endogenous modulation of essential neural lipid components which together with cholesterols are involved in the induction of normal motor and mental capacity. The 100% increase in the use of psychoactive medicines during the last decennium, the tripling of sleepingpill consumption in five years, and pain-killer medicines may also mirror the deficient intake of essential vital lipids, leading to disparate neurological disorders [4, 5]. Sterile inflammatory diatheses [6] like, inflammatory bowel disease (IBD), post traumatic stress disease (PTSD) and belong to these ailments signalling the nerves' distress by the pain sensation as a warning that they cannot produce the essential neural lipids without supplementation, required to regain mental and motor function. They also lack the lymphopoietic titanium containing CNS-lipid factor mandatory to sustain a normal immune response [7, 8]. This aberration in the immune system may then lead to different chronic sterile inflammatory afflictions, Table 2. One of the most serious sterile inflammatory diseases is pancreatitis. Calcium (Ca) activates the phospholipase A2 enzyme which splits arachidonic acid in lecithine into lysolecithin. The inflammation proceeds dangerously if the calcium ions in the patients' serum are not chelated by CaNa_2_ EDTA to inactivate the phospholipase_A2_ whereby the neutralisation of acute pancreatitis can biologically be cured in some days [7, 9].

## 3. The Effect of Dietary Supplementation with Central Nervous System (CNS) Lipids 

The positive clinical effect from feeding ASD-afflicted children with (CNS) lipids supports the notion that these vital lipids are taken up by nerve tissues from the blood circulation and can thus alleviate central and autonomous nervous system illness. The amount of the dietary supplement of CNS lipids was prescribed as 10 g × 2/day, measured as two teaspoons of tinned healthy piglet or reindeer brain (Neurofood Ltd. containing 220 g/can) mixed in a drinking glass of cold lemon juice, to shield the taste. Alternatively, a special lemon ice-cream with the lipid content (10%) based on CNS-lipids, to replenish the cream (produced by Ingman Foods Inc., for Neurofood Ltd) was fed twice a day, in two ready-made portions of 100 g ice-cream “*N-Ice*”, containing 10 g CNS-lipids. The dose-level requirement is individual, and usually metered in the child as a decrease in hyperactivity and improved mental balance. The ASD patients' general mental improvement, decreased hyperactivity, and better learning functions could be recorded in a surprisingly short time, measured in weeks from the start of a regular oral intake of CNS-lipids. The intake of these natural vital lipids cannot harm the patient! 

Anyhow, we should clearly distinguish between the *unhealthy* effects implicated with ingestion, or production of energy fats from, for example, carbohydrates, wheat, lard, margarine, fried food, rybe seed oil, and transfatty acids which humans should avoid, but animals gather before winter as a subsistence reserve, as compared with the *healthy* intake of vital lipids, butter, cream, brain, sweetbread, liver, fatty fish, eggs, and nuts representing the building blocks required by the CNS to uphold normal harmonious synapto-genesis. A dietary prion-free CNS-lipid supplement can also palliate other concurring neurologic deficiency syndromes in adults like, burn-out, chronic fatigue syndromes, post traumatic stress disease PTSD [10], insomnia, neural pain, vomiting, vertigo, hyperesthesia, restlessness, soft tissue rheumatism (without elevated Rh factor), and stiffness, which may have a similar aetiology as ASD.

## 4. The Effect of Our Changed Dietary Habits

The dramatic change in our dietary habits during the recent decades, linked to the provoked and unnatural fear of all forms of dietary lipids, aggravated by consumption of statins and cholesterol depressing “fat-free” diets may result in an unhealthy metabolic deficiency encompassing the availability of essential lipid molecules in the maternal blood circulation during the first trimester when the foetal brain and spinal cord are induced [11]. Her breast milk which is especially rich in lipoproteins, lipids with sugar, and trace-element ions secures the healthy development and intelligence (IQ) of her offspring. This induced alimentary deficiency in the pool of vital lipids may cause the mother unwillingly to be the culprit for ASD, and not her child [4]. 

To analyse this hypothesis a special questionnaire was devised for mothers of children suffering from the seemingly mysterious “autism spectrum disorder,” presently diagnosed as a disturbing incidence of Autism and ADHD, as well as related to other mysterious neurological afflictions like decreasing the risk for postpartum psychosis. The questionnaire analysed dietary habits of the mother, the possible presence of a wide panel of neurological perturbations linked to the central nervous system, and/or her autonomous neural functions. In previous studies it was found that neural viral infections (e.g., herpes) could cause a blood-brain barrier lesion letting CNS lipids to flow into the patients' blood, a lesion associated with depressed lymphocyte function [8], which could be compensated by ingesting well prepared brain (100 g/week). Suggestively, it replenished a loss of a lymphopoietic CNS-lipid factor containing titanium (Ti), a collateral to the cobalt (Co) in B_12_ for erythropoiesis. Alternatively it furnished essential CNS building-blocks for processing by organ-specific mitochondria [7, 12] exclusively present in the special endothelial cells of brain capillaries [11, 13], which are responsible for compensating, during our sleep, the daily consumption of vital lipids caused by bodily activities and stress.

## 5. Findings with a Questionnaire, Analysing Neurological Disorders in Mothers of ASD Children

In twelve mothers of children suffering from ASD all had adhered to *fat-free diets* during pregnancy. All but one had suffered from intense *vomiting* during the gestation ranging from two to nine months. One mother had vomited profusely in the beginning of her two previous pregnancies, but with her third, when she ingested CNS lipids she did not even know that she was pregnant before her hormone tests revealed it. The lack of circulating vital lipids in early pregnancy may force the body to draft CNS lipids required for the induction of the foetal brain and the spinal cord from the autonomous nervous tissue of the mother. This physiologic depletion in the autonomous neural system may then lead to vomiting in the morning, as a form of cramp. 

Some had suffered from *stress, depression, sleeplessness, pain, and stiffness, *that is, functional aberrations we have found to be linked to deficiencies in vital lipids. This postulation is backed by the correcting effect obtained by feeding patients these natural vital lipids. Nine of the mothers had previously experienced *herpes virus* infections, two *postpartum depression* and difficulties with breast-feeding. 

The effect of oral administration of prion-free CNS-lipids, prepared according to a good French recipe, or mixed with fruits using canned brain (Neurofood Ltd. Helsinki), fed to mothers, (100 g/week) decreased pain and mitigated their depression. When given this to thier children suffering from ASD this supplement (70–100 g/week) decreased children's restlessness in only three weeks. If the regular intake of vital lipids was stopped the child got a relapse registered as intense restlessness. When the special diet was reinitiated a more normal mental balance was regained. Feeding a 20-year-old boy, severely autistic since childhood, with CNS-lipids in the form of healthy piglet brain mixed with assorted fruits recuperated him in 1.5 year to such an extent that he got a job, which he still holds.

Neural pain in cancer is regarded as a warning signal from nerves emanating from a lack of endogenous vital lipids required by the CNS. When supplied by feeding these essential lipids start to circulate in the blood and the nerve tissue can integrate them, thus turning off the distress signal—it can act as a strong physiologic analgesic. The effect is linked to individual dose requirements. These clinical results give a further support to the hypotheses that one major aetiological factor for ASD is a lack of vital lipid molecules required in embryogenesis, and normal induction of the foetal CNS [5]. 

As a by-line, in preliminary trials with adults, various other neurological clinical symptoms like burn-out, fibromyalgia, and Crohn's and Reiter syndromes were alleviated following dietary supplementation with CNS-lipids in synergy with certain amino acids. The lymphopoietic factor in CNS-lipids seemed to be an important curative cofactor [6, 13] to palliate these chronic sterile inflammatory diseases.

## 6. Embryogenesis and Normal Induction of CNS Require a Constant Dietary Supply of Vital Lipids

We have to discern between the healthseffect of ingesting energy fats and that of ingesting beneficial vital CNS-lipid molecules. Storage of energy fats in our organs is unhealthy, but vital lipids shape the billions of different cognitive lipid molecules forming 70% of our brain tissue mass, securing its normal mental network, leading to harmonious synaptogenesis. A constant minimal natural supply of these metabolic vital lipid building blocks or their CNS precursors seems to be necessary to avoid neuro-developmental disorders. Table 1 gives a short list of CNS “lipidomic” functions. 

To secure normal mental capacity a dietary supplementation with vital lipids is required throughout our whole lifespan. In embryogenesis the brain and spinal cord, with their high lipid content are induced during the first trimester necessitating therefore a pool of circulating maternal functional lipidsoluble substances. These are essential to cope with the physiologic requirement, and CNS reconstitution. 

Humans also have rapid brain growth for about two years after birth, with early postnatal development at a rate of 250,000 neurons per minute. Until puberty there is a constant increase of neurons, axon diameter and myelination representing the important continuation of brain maturation into adulthood. This constant development of “hypermorphosis” in humans, leads to a brain weight to body weight 3.5 times that of apes, by the time we are full-grown [11]. This prodigious extended neuron production, over the 20 months after birth, seems to indicate that our “gestation”, may be considered to last for more than two years [5, 11, 13]. Further, new neurons are still formed in the adult brain especially if it is constantly mentally trained.

## 7. Specific Mitochondria Are Exclusively Present in the Endothelial Cells of Our Brain Capillaries

Following the human genome project a surprising nucleotide sequence analogy between species was found. The human chromosomes were 99% identical with monkeys, 96% with rats, and 60% with flies. If evolution was based solely on random mutations such a high nucleotide analogy would not exist! 

Mitochondria are endowed with many functions, organ-specific mitochondria seem to activate neural stem cells' to furnish the cell-nucleus with energy, regulate gene transcription [14], and can repair mutations. The only explanation for the nucleotide analogy is that mitochondria with their special mtDNA must have been involved in building the chromosomal structures, over eons of their phylogenetic toil [15, 16] actually forming in them, *the memory of evolution*. 

Interspecies nucleotide analogies suggest that mitochondria represent a primary function in evolution, with Darwinism signifying an important but secondary selection system. An indication of the central position of mitochondrial mtDNA could lead them, during embryogenesis, to modulate the 200 different organ-specific mitochondria required to control all our 200 special tissue cells, in a healthy body. *Brain capillaries have numerous mitochondria*, which other tissue capillaries lack [13]. 

As shown, organ-specific mitochondria are gene regulatory and can prevent experimental leukaemia induction in rats [12]. Transformed mitochondria are also seen to force back human malignant cells to normal healthy transcription without apoptosis [17]. They seem to have a memory of what they have constructed. In Arabidopsis plants identical mutations present in both chromosomes can be corrected, surprisingly evidenced as 10% of the offspring of these plants were healthy [14]. This unanticipated result does not require a need to change Mendel's Law, since it simply suggests that mitochondria have a memory of the nucleotide sequence in an organism they have created during evolution. They can therefore detect this aberration during replication and correct the mutation in their chromosomes. Organ-specific mitochondria are most likely also involved in activating autologous new specific tissue cells, as seen with human foetal skin transplants curing severe skin burns in children [18]. Human skin transplants cultured from a male foetus could cause autologous skin to grow in a female recipient. The explanation seems to be that these foetal transplants must have contained tissue-specific skinmitochondria in order to be able to be cultured and induce new autologous skin cells. Organ-specific mitochondria may thus have transgressed the recipients cells and specifically activate the skin genes present in the chromosomes of any tissue cell. The activation of the recipients skin cells precludes rejection and represents a step ahead of stem cell activation. Present stem cell techniques are maimed by a lot of constrains.

Similar organ-specific mitochondria, exclusively present in the endothelial cells of our brain capillaries, seem to modulate the vital circulating metabolic lipids fed, required as CNS-lipid precursor molecules, to compensate during our sleep the natural vital lipid consumption caused by stress and daily activities. We sleep to provide a physiologic interlude for our CNS to sustain mental balance and good motor function. 

Brain capillaries with their special interconnected endothelial cells form tight junctions [13], which let through only lipids or lipid soluble components into the brain, but exclude transferral of many toxic substances. These modulated, schooled active CNS factors, required to compensate the natural daily consumption seem to be shaped by the specific endothelial capillary mitochondria, thanks to the oral intake of risk-free lipid precursor factors forming brain lipids. These are then further processed during our sleep to make us ready for active work. The transfer of the formed functional lipid complexes to the brain substance is processed through the tight junctions, forming the blood-brain barrier. With brain lipids contained in the diet all the billions of lipids are replenished, constructing all the vital lipids forming the functional CNS “*lipidome*,” which together with *genomics* and *proteomics* acts to sustain health. This vital lipid requirement is not only dependent on furnishing CNS with DHA (docosahexaenoic acid), PUFA (polyunsaturated fatty acids), and 3-omega [19], these and their precursors are actually all contained in the specific diet prescribed, and required as essential physiological neural components. The body is agile to selectively accrete any missing lipidmolecule, precursor factor, cholesterol or choline to form and sustain a functional CNS. This supplementation also integrates natural essential trace-element ions [20], the specific brain aminoacids, tyrosine & tryptophan, plus B_ 12_ and folic-acid vitamins, into the CNS [11]. This nutritional augmentation is again designed to compensate the daily mental consumption of these lipid factors. Active transfer of vital lipids to the brain substance is mediated by receptor-mediated endocytosis [13], among other systems. Prolactin may also be involved in producing progenitor nerve cells [11]. One remarkable feature in the induction of brain, for which the Hedgehog signal transduction pathways is important, is that it needs cholesterol, since this is critical for the catalytic cleavage of Sonic Hedgehog protein [11], derived from the notochord, to sustain nerve induction. 

Any minute lipid deficiency [4] or even more intricate combined dietary faults during any phase of gestation may affect the programming of this complex multilayer synaptic network. A fault in the timely induction at any level in this network can therefore explain the presentation of this disparate seemingly incoherent spectrum of mental symptoms in ASD patients. This variation in the expression of ASD has occasionally even been given specific names as, Tourette or Asperger syndromes although they may have the same aetiology. There is possibly no need to search for other causative factors.

## 8. Vital Lipids Seem Also to Be Involved in the Inductional Signal System Regulating Allergic Reactions

In studies with horses suffering from a seasonal skin allergy “sweet itch” [20] a surprising healing reaction was found by diluting the patients serum tenfold followed by vigorous shaking (succussion 100 times) repeated three consecutive times. Some drops taken from the upper floating part of the mixture could surprisingly cure the allergy in horses. This “homeopathic-like” factor could be an inductional CNS-lipid component excreted into the patients' blood in excess and thus aggregate and be too big to fit its cell-receptors. These would be the only biological factors which could avoid dilution in this procedure, since succusing the affected serum vigorously will emulsify the lipid components present in the diseased animals' serum and as it floats actual dilution is prevented. The last tenfold dilution was made in 50% alcohol. A logic explanation for the supernatants curative effect excludes an effect from any chemical or peptide component since the dilution became too great. But if the signal effect was based on inductional CNS-lipids which primarily had been excessively excreted, they spontaneously formed big aggregates, too big to attach to pertinent lipoprotein cell-receptors. The last dilution in alcohol (50%) could dissociate these vital lipids into monomers which then could attach to their cell receptors and revise the allergic reaction. Studies with diluted horse sera suffering from “sweet itch” (10^−6^th) and that of healthy controls have been continued for 15 years with over 200 randomly selected horses treated. Approximately 75% of the horses have been cured. 

Professor Madelene Ennis reproduction of the histamine-like reaction on white cells may have been elicited by the effect of a cell receptor for histamine, and not from the histamine proper.

## 9. Conclusion

During normal gestation, the mothers' immune defence is spontaneously improved to defend her embryo [21]. Furthermore, her hormonal balance and the whole maternal dietary metabolism are closely implicated in normal embryogenesis [1, 21]. Presently, there seems to be a valid suspicion based on clinical pilot studies that ASD in children, as well as certain related neurological disorders in adults, stems from a lack of circulating nutritional neural lipids. Therefore, we may have an urgently needed opportunity to decrease the incidence of ASD, as a neurodevelopmental fault triggered in children, by ethically recommending healthy females simply to ingest, before and after pregnancy—and during breast feeding—a risk-free diet containing vital lipids like; butter, cream, chickenliver, salmon, nuts, sweet bread, and especially cooked *prion-free brain*, to secure normal mental development of her offspring, and a satisfac-tory development of the IQ in her child. Breast milk also contains vital CNS-lipid precursors and is therefore so important for the young child during its early postpartum development.

## Figures and Tables

**Table 1:** Lipidomics, the function of billions of vital lipid molecules forming our brain and spinal cord.

(a) Embryogenesis, with organ-specific mitochondria leading to harmonious synaptogenesis.	
(b) Consciousness, intellect, fantasy, memory, intuition, experience and telepathy.	
(c) Inductional CNS is linked to cancer control and healthy gene transcription.	
(d) Blood-brain barrier lesions upset the axonal inductional cell control (e.g., herpes virus infections).	
(e) Lymphopoietic stimulation by a CNS lipid molecule containing titanium (Ti), as cobalt in B_12_.	
(f) Neurological pain, the warning signal from nerves is alleviated by ingestion of CNS lipids.	
(g) Burn-out stress syndromes in sportsmen, battle fatigue, and sleep disorders eased from CNS diets.	
(h) Rheumatic, neuropathic, psoriatic arthritis, fibromyalgia, Crohn's disease, and IBD, PTSD respond to CNS diets.	
(i) Idiopathic neural pain, sterile inflammatory diatheses, and insomnia are mitigated by dietary CNSlipids.	
(j) Marked regional depletion of CNS-lipids found in spinal cord segments may link it to atherosclerosis.	
(k) The aetiology of autism spectrum disorder (ASD) and ADHD seems to be due to a deficiency in circulating vital CNS-lipids during gestation and after birth.	
(l) CNS-lipid monomers may be involved in controlling allergic reactions via cellreceptors.	
(m) Melanoma satellites appear in the enervated axonal area—affected by impaired CNS induction.	
(n) Cholesterol levels dip from ingestion of extracts made from brainlipids and membranes.	
(o) A prolonged relative lack of vital CNS-lipids may cause anorexia and infertility.	
(p) Mitochondrial lesions lead to derangements in the lipidome.	

**Table 2:** Biological dietary treatment for various sterile inflammatory diatheses.

In cancer studies based on a combined biological and specific stimulation of the patients immunity to autologous tumour markers (bioimmunotherapy) a lymphopoietic central nervous system lipid (CNS) component was found to be crucial for normal white cell function [1]. A deficiency in this vital CNS-lipid component can also cause aberrant inflammatory reactions, as in Inflammatory bowel disease (IBD), and in several other related sterile inflammatory diatheses e.g., crohn's disease polyarthritis. This CNS lipidfactor, containing titanium (Ti), is essential for a normal immune response (as a collateral to the cobalt in B_12_ for erythropoiesis). The high and soaring incidence of these sterile inflammatory diseases is causing a major general health problem while lacking effective curative treatments. In bioimmunotherapy for colon cancer, since the seventies, the precancerous Crohn's disease (CD), and ulcerative colitis (UC) were found to be alleviated and cured following specific dietary supplementation. The aim was therefore to try to delineate these nutritive factors and to uncover effective, sustainable biological treatment modalities for these diverse chronic sterile inflammatory diatheses since they are potentially precancerous, like any chronic inflammation.	

Nutritional supplementation acting as functional foods, containing a physiologic combination of: certain amino-acids, trace-element ions, vital central nervous system (CNS) lipids, the lymphopoietic lipid component containing Ti, plus physiologic amounts of vitamins, especially B_12_ were, fed as ready-made powders, to improve patient compliance, in preliminary therapeutic trials for; CD, UC, fibromyalgia (FbM), rheumatism (Rh) lacking increased Rh factor, psoriasis (PSO), polyarthritis (PyA), periosteal bone pain (PP), pancreatitis (PaC), Reiters disease (RD), and so forth.	

These aberrant inflammatory reactions could in certain patients be alleviated by nutritional biotherapy administering-specific aminoacids (2–5 g/d) mixed with trace-element ions; Cr, Mn, Rb, Se, Sr, V, W (1.5–2.5 mg/d) and CNS-lipids, with assorted fruits as a tasty blend, easily prepared using canned healthy prion-free brain lipids, produced by Neurofood Ltd. Finland. Canned brain containing 220 g is sufficient for two weeks as a dietary supplement furnishing the essential CNS lipids our healthy body requires. The dietary supplement of amino acids required is Leu, Lys, Arg, and also Gly, Glu, (to prevent splitting of the glutathione in the leukotriene molecule by *γ*-GT into Glu, and Gly whereby *γ*-GT is inhibited (also by boron). This results in substrate inhibition and can thus prevent the leukotriene-cascade cycle causing sterile inflammatory lesions, expressed as increased capillary permeability, and formation of the slow-acting substance of anaphylaxis. These organic and inorganic alimentary supplements ingested in addition with 220 g of CNS lipid molecules (every second week) have caused a positive clinical response. Dietary supplements could mitigate symptoms of CD in some weeks, and the clinical effect could be sustained for years. Biotherapy for UC is principally linked to the same dietary formulation, with the further addition of, Asp Ile, Ser, Thr, (5 g/d), + Mo, Se, and W (1.5–2.5 mg/d) + CNS-lipids. For FbM mainly, Gly, Glu, Leu, Arg, + all trace-element ions mentioned, and CNS-lipids mixed in fruits for the sake of taste; for Rh, Ala, Ile, Leu, Ser, The trace-element ions, + CNS-lipids. For PSO especially Ile, Gly, Glu** + **Tinn (Sn) and CNS-lipids. In PYA, PP, supplying especially strontium (Sr) and serine (L-Ser) is essential to keep bone membranes (periost) healthy. PaC requires a lowering of serum phospholipase-A activity. This can be achieved by chelation of the serum calcium ions, which activates this lipidsplitting enzyme, involved in causing pancreatitis.	

Beneficial nutritional clinical therapy for these diverse premalignant diatheses is dependent on normalization of the immunesystem in patients by ingesting this lymphopoietic CNS-lipid component (forming the functional “lipidome” based on billions of vital CNS-lipid molecules) acting in concert with certain essential amino acids and trace-element ions regulating healthy cell induction. Dietary supplementation is curative inexpensive, and causes no sideeffects.	
